# Fabrication and Microstructure of ZnO/HA Composite with In Situ Formation of Second-Phase ZnO

**DOI:** 10.3390/ma13183948

**Published:** 2020-09-07

**Authors:** Shidan Yuan, Ye Ma, Xingyi Li, Zhen Ma, Hui Yang, Liting Mu

**Affiliations:** 1 School of Materials Science and Engineering, Jiamusi University, Jiamusi 154007, China; jmsdxysd@163.com (S.Y.); 15046492438@163.com (Y.M.); mz252930179@163.com (Z.M.); freedomyahu@163.com (H.Y.); muliting@163.com (L.M.); 2 School of Pharmacy, Jiamusi University, Jiamusi 154007, China

**Keywords:** powder sintering, grain growth, compression strength, ZnO/HA composite

## Abstract

Nanometer hydroxyapatite (n-HA) powders were synthesized by the chemical precipitation method, and a novel ZnO/HA composite, which consisted of second-phase particles with different sizes and distributions, was successfully fabricated. ZnO/HA composites were prepared by using powder sintering with different Zn contents and a prefabrication pressure of 150 MPa. Microstructure and local chemical composition were analyzed by a scanning electron microscope (SEM) and energy-dispersive spectrometer (EDS), respectively. The phase composition and distribution of the composite were determined with electron back-scattered diffraction (EBSD) and an X-ray diffractometer (XRD), respectively. The experimental results of the XRD showed that the chemical precipitation method was a simple and efficient method to obtain high-purity n-HA powders. When the sintering temperature was lower than 1250 °C, the thermal stability of HA was not affected by the Zn in the sintering process. Due to sintering in an air atmosphere, the oxidation reaction of Zn took place in three stages, and ZnO as the second phase had two different sizes and distributions in the composites. The compressive strength of ZnO/HA composites, of which the highest was up to 332 MPa when the Zn content was 20%, was significantly improved compared with pure HA. The improvement in mechanical properties was mainly due to the distribution of fine ZnO particles among HA grains, which hindered the HA grain boundary migration and refinement of HA grains. As grain refinement increased the area of the grain boundary inside the material, both the grain boundary and second phase hindered crack development in different ways.

## 1. Introduction

Hydroxyapatite (HA, Ca_10_(PO_4_)_6_(OH)_2_) has been widely considered to have good biocompatibility. There are many methods to synthesize HA, such as dry methods [1,2], the chemical precipitation method [3], and hydrothermal method [4]. The HA powder materials prepared by the above methods have been widely applied in many fields, such as bone filling repair [5], the tissue engineering stent [6], the implant surface coating [7], drug delivery [8], and environmental pollution treatment (such as wastewater treatment) [9]. The theoretical density of HA is 3.16 g/cm^2^, and a simple and effective method to prepare HA block materials is by pressing HA powder and sintering it at high temperature.

The mechanical strength and elastic modulus of dense HA are higher than those of human bone except for the fracture toughness, which cannot meet the needs of bone tissue repair and replacement of human load-bearing parts; and HA with a larger grain size and higher densification degree has good biocompatibility but lower bioactivity. In order to solve such problems, many scholars have carried out a lot of research works. The preparation of porous hydroxyapatite materials is a hot spot. The porous structure with a different morphology, size, and distribution in porous HA could provide the necessary space to ensure the transmission of blood and other substances, thereby ensuring the metabolism of bone tissue. At the same time, the high specific surface area would be more conducive to the interchange of materials between the implant and human tissues, which could accelerate the formation and reconstruction of new bone tissues [10]. However, the porous structure has a great influence on the mechanical properties of materials (such as strength reduction). HA composites are also another research hotspot. Combining two or more materials (including HA) would give full play to their respective advantages and produce a synergistic effect to meet the needs of different applications. The composite with HA now mainly includes inorganic and polymer materials [11,12], which must meet the requirements of good biocompatibility and excellent metabolic ability of the human body. Zn is an important trace element in the human body involved in the process of nucleic acid protein metabolism, and can also promote the normal development of skin, bone, and sexual organs, and maintain digestion and metabolic activities [13,14]. ZnO can be used as a source of Zn elements for the human body. Studies have shown that ZnO with small size is an ideal non-polar antibacterial agent with broad-spectrum antibacterial effects [15,16]. Based on several reports, the ZnO/HA composites, prepared by using ZnO as a second phase or HA being modified with ZnO, still had broad-spectrum antibacterial properties [17]. However, a series of problems have been found during the preparation and clinical application of HA composites, such as the uncontrollable grain growth for HA and ZnO during sintering, low or no antimicrobial properties, and unsuitable mechanical properties for human bone tissue, which limit the application of HA composites. Few reports have focused on the grain growth for porous HA ceramics prepared with Zn powder as the pore-forming agent. Therefore, the biomedical hydroxyapatite ceramic materials with a specific shape and used as load-bearing parts for the human body should be developed in the porous and multi-functional direction.

In this study, spherical Zn particles were used as a pore-forming agent. The oxidation reaction of Zn powder and oxygen was used to obtain the second phase of composites. The gaps between HA nanocrystals were used to control the size of the second-phase particles. The ZnO/HA composites with porous structure were prepared by conventional pressing and pressureless sintering, aiming to reduce the compactness of the material and ensuring the mechanical strength of the material simultaneously. The densities of the material, as well as the size and distribution of ZnO particles, were controlled by adjusting the addition amount of the Zn powders and the sintering process. The relationship between the phase composition, microstructure evolution, and mechanical properties of the composites was analyzed and discussed.

## 2. Materials and Methods

Calcium hydroxide powder (purity 95.0 wt.%, Tianjin Guangfu Fine Chemical Research Institute, Tianjin, China), calcium dihydrogen phosphate monohydrate (purity 98.0 wt.%, Tianjin Guangfu Fine Chemical Research Institute), and zinc powder (purity 99.5 wt.%, −400 mesh, Tianjin Ruijin Chemical Co. LTD, Tianjin, China) were used in this study as raw materials. The experimental equipment for the preparation of pure HA and ZnO/HA composites are listed in Table 1.

### 2.1. Preparation of Nano-HA Powders, Pure HA, and ZnO/HA Composites

**Figure 1:**
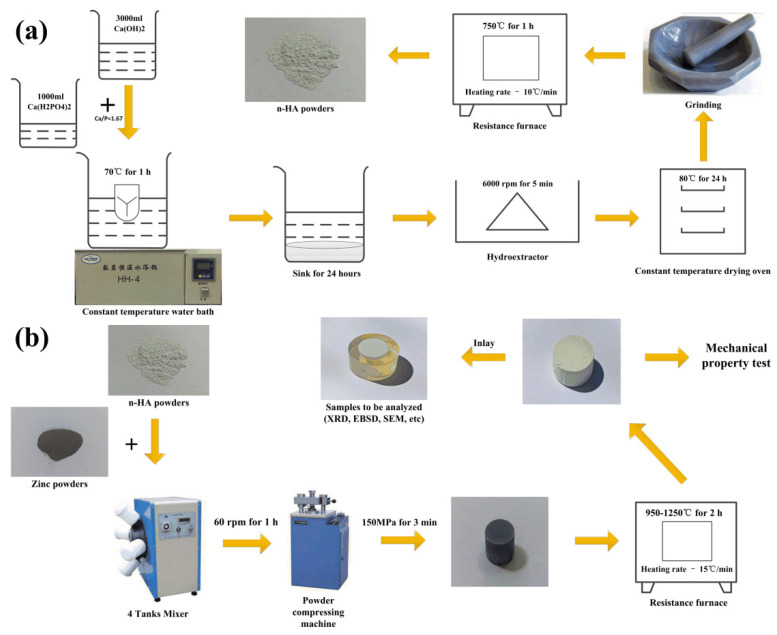
Preparation process of (**a**) nanometer hydroxyapatite (n-HA) powders and (**b**) ZnO/HA composites.

The nanometer hydroxyapatite (n-HA) powders were prepared by the chemical precipitation method. The technological processes are shown in Figure 1a. Pure Zn was used as the pore-forming agent. According to the mass ratios of 10:0, 9:1, 8:2, and 7:3, the n-HA powder and Zn powders were mixed in the mixer. Pure HA and novel in-situ ZnO/HA composites were prepared by sintering at different temperatures after pressing at 150 MPa (Figure 1b). The composition and sintering process parameters for the preparation are shown in Table 2.

### 2.2. Compression Strength Test

Compressive strength was evaluated using a mechanical property testing system at room temperature and with the loading speed of 0.5 mm/min. The composite samples with a diameter of 10 mm and thickness of 6 mm were prepared in the same manner.

### 2.3. Characterization

Microstructure and local chemical composition were analyzed by a scanning electron microscope, SEM (JSM-7800F, JEOL LTD, Tokyo, Japan), and energy-dispersive spectrometer, EDS (Oxford X-maxn, Oxford Instructments, London, UK), respectively. The phase composition and distribution of the composite were determined with electron back-scattered diffraction, EBSD (Oxford Nordlys Max3, Oxford Instructments, UK), and an X-ray diffractometer, XRD (Bruker D8, Karlsruhe, Germany), by using CuKα radiation (λ = 0.1542 nm), and using a vertical goniometer in the 2θ range of 10–100°, with a step of 0.02° and at a scan speed of 5° min^−1^.

## 3. Results and Discussion

### 3.1. Characteristics of Synthesized Nano-HA Powder

**Figure 2:**
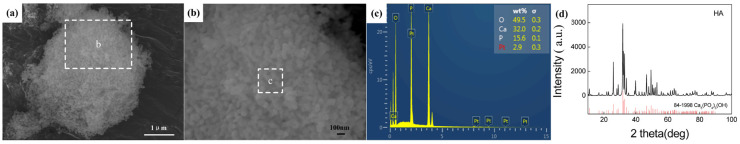
(**a**,**b**) SEM morphology; (**c**) EDX spectrum, and (**d**) XRD pattern of n-HA powders.

The morphology, chemical composition, and phase analysis of the n-HA powder sample synthesized by the chemical precipitation method are shown in Figure 2. A homogeneous distribution of particles was obtained after crystallization treatment at 750 °C, and there was a slight agglomeration phenomenon after drying (shown in Figure 2a). Figure 2b shows an enlarged SEM image of a part of the area in Figure 2a, and it can be seen that the grain size is between 40 and 100 nm. The EDS analysis results of region C in Figure 2b is shown in Figure 2c. It has been shown that the chemical components are Ca, P, and O (H element cannot be detected) and the molar ratio of Ca to P is about 10:6. The results of XRD indicate that n-HA powders have a high degree of crystallization, as shown in Figure 2d. The diffraction peaks have a certain width, which corresponds to the nanocrystalline morphology of the powders. The phase analysis has shown that it is HA (Ca_5_(PO_4_)_3_(OH), JCPDS #84-1998), and no other calcium phosphate phases exist. The experimental results have shown that n-HA particles, which are synthesized by the chemical precipitation method, have high purity.

### 3.2. Characteristics of Pure HA and ZnO/HA Composite Samples

**Figure 3:**
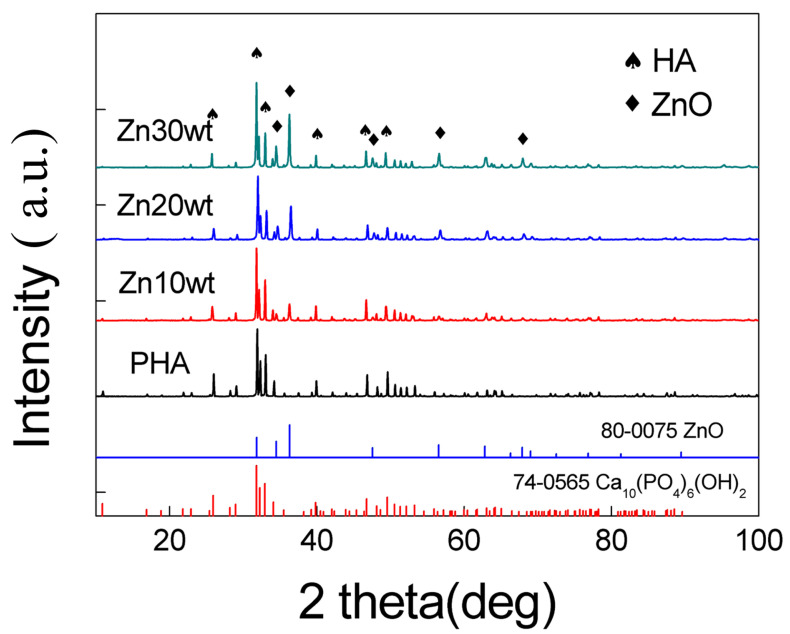
XRD patterns of the ZnO/HA composites.

The phase compositions were characterized by XRD. The results have been shown in Figure 3, which indicate the results of the composites with different contents of Zn at the sintering temperature of 1250 °C.

The experimental results have shown that the ZnO/HA composites consist of two phases, (HA (Ca_10_(PO_4_)_6_(OH)_2_, JCPDS #74-0565) and ZnO (JCPDS #80-0075)). As the sintering atmosphere is air, Zn in the sintering process oxidized with the oxygen in the air to form ZnO. The relative intensity of the ZnO diffraction peak increased with the Zn content. It has been shown that three strong peaks of HA and ZnO correspond well to their respective standard cards, and the positions of the diffraction peaks are not shifted either. These results show that HA did not decompose with the presence of zinc in the sintering condition of 1250 °C [18,19,20]. They also show that the thermal stability of HA was not affected by the presence of elemental zinc and zinc oxide when the composite was sintered below 1250 °C.

**Figure 4:**
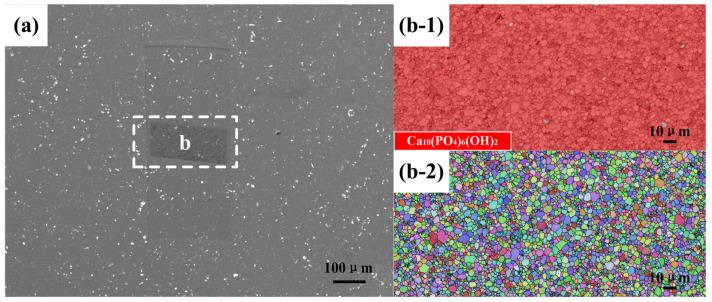
(**a**) SEM morphology; (**b-1**) phase distribution; and (**b-2**) inverse pole figures of pure HA sintered at 1250 °C.

The EBSD technique combined with SEM has been used to analyze the crystal structure and morphology of pure HA and ZnO/HA composites. The SEM morphology of pure HA samples sintered at 1250 °C is shown in Figure 4a. The experimental results show that there were a small number of cavities with different shapes and sizes ranging from 0.3 to several μm, which were distributed in the polished samples. HA grain growth was caused by grain boundary migration in the sintering process, and the grain growth represents the reduction in internal voidage of the material at the same time, which increased the densities of the material [21]. The cavity size gradually decreased with grain boundary migration. When the HA grain orientation around the cavity had a large angle grain boundary and the boundary could not move toward the interior of the other grains, the HA grain growth around the cavity ended. In order to confirm the phase distribution, EBSD analysis was performed for region b in Figure 4a. As shown in Figure 4b-1, it is a phase (PH) distribution superposed with band contrast (BC), in which red represents the HA phase, gray represents a small amount of zero-resolution phase, and black corresponds to the cavities in Figure 4a. The inverse pole figures (IPF) of region b in Figure 4a is shown as Figure 4b-2, which is superimposed with grain boundaries (GB) and BC graphs, where different colors represent different crystal orientations. According to IPF, the HA grain orientation distribution should be random and have no special direction.

**Figure 5:**
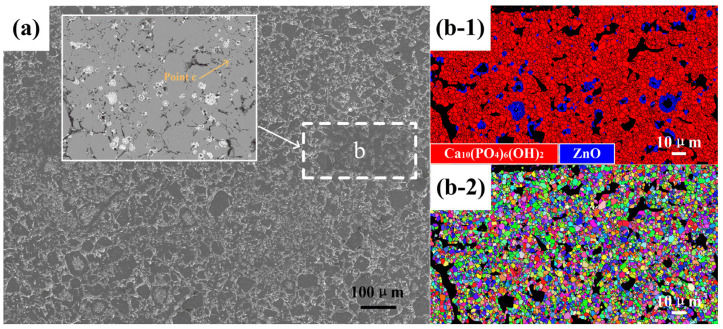
(**a**) SEM morphology; (**b-1**) phase distribution; and (**b-2**) inverse pole figures of ZnO/HA composite sample with 10% Zn addition sintered at 1250 °C.

The SEM morphology of the ZnO/HA composite with 10% Zn addition sintered at 1250 °C for 2 h is shown in Figure 5a. It can be seen from the figure that a certain number of cavities with different shapes and sizes were distributed inside the materials. There was a certain number of cavities of nearly spherical shape also distributed, except for the cavities described in Figure 4a above. EBSD analysis on region b in Figure 5a has been shown as Figure 5b-1, and the phase distributions are superposed with BC, in which the red area represents the HA phase, the blue area represents the ZnO phase, and the black area corresponds to the cavities of different shapes and sizes in Figure 5a. The IPF of region b in Figure 5a is shown as Figure 5b-2, which is superimposed with GB and BC graphs, where different colors represent different crystal orientations. From the IPF, it can be preliminarily determined that the HA and ZnO grain orientation distribution was random and had no special direction. The density of the material changed slightly during the heating period, and the density of the composite was still at a low level. With the increase in temperature, the reaction rate between Zn and O_2_ accelerated as the interior of the material remained connected with the air atmosphere, which promoted the formation of a certain thickness of ZnO on the surface of spherical Zn particles. The layer of ZnO acted as a coat to isolate the internal Zn from the external oxygen and prevented further oxidation of the Zn. When the temperature rose further beyond 419.5 °C (the melting point of Zn), the unoxidized part inside the spherical Zn particle changed from solid to liquid. Part of the liquid Zn flowed out of the gaps in the zinc oxide protective layer and dispersed into HA intergranular gaps around the Zn particles. Several fine-sized ZnO particles (such as Point c) formed by the continuous oxidation reaction between liquid Zn and O_2_. When the temperature exceeded 907 °C (the boiling point of Zn), the residual liquid Zn in the Zn particle boiled and spilled out from the inside of the sample, and part of the Zn atoms oxidized and remained in the sample. The other part flew off the surface and oxidized.

**Figure 6:**
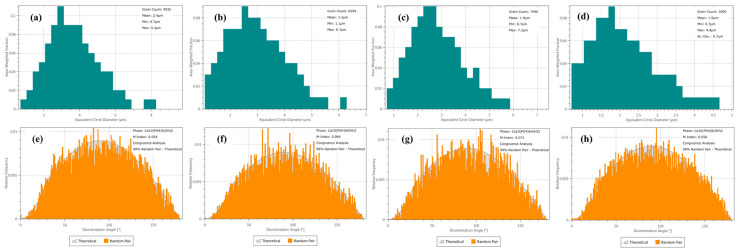
Grain size of HA and orientation distribution ofpure HA and ZnO/HA composites: (**a**,**e**) Pure HA; (**b**,**f**) Zn 10 wt.%; (**c**,**g**) Zn 20 wt.%; (**d**,**h**) Zn 30 wt.%.

The relevant grain size and orientation distribution information were obtained by processing with Aztec Crystal software, according to the original EBSD data of ZnO/HA composites. Distribution of the grain size and orientation, which describes the equivalent circle diameter, is shown in Figure 6. The experimental results show that the HA grain orientation was randomly distributed in the material, shown in Figure 6d–f. The average equivalent circle diameter of HA grains in pure HA and ZnO/HA composites, which have been prepared with 10%, 20%, and 30% amounts of Zn, were 2.4, 2.2, 1.9, and 1.5 μm, respectively, shown as Figure 6a–d, and they were normally distributed.

**Figure 7:**
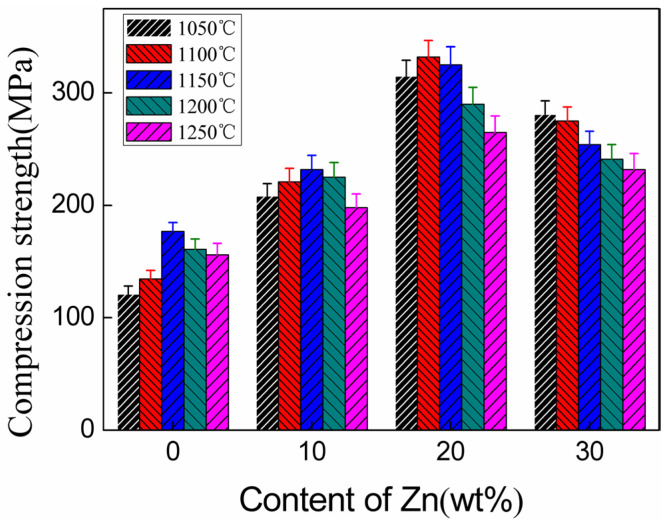
The compression strength of pure HA and ZnO/HA composites.

As shown in Figure 7, it is found that the compressive strength of the composites is higher than that of pure HA sintered with the same temperature. Experimental results have shown that the compressive strength of the composites increased first and then decreased with the increase in Zn addition, and the compressive strength reached the maximum value when the addition of Zn reached 20% as well. Compared to the composite samples with the same Zn content but different sintering temperature, it is found that the trend of compressive strength changed. It has also been found that with the increase in sintering temperature, the compressive strength of the composites increased first and then decreased, and the maximum compressive strength appeared at 1150 °C when the contents of Zn were low. With the further increase in Zn contents (20% Zn, 30% Zn), the maximum of compressive strength was offset in the low-temperature zone (20% Zn corresponds to 1100 °C, 30% Zn corresponds to 1050 °C). The results showed that an oxidation reaction occurred between Zn and oxygen in air to form ZnO during sintering under the condition of air atmosphere, and ZnO existed in the composite as a second phase, which affected the compressive strength [22,23].

**Figure 8:**
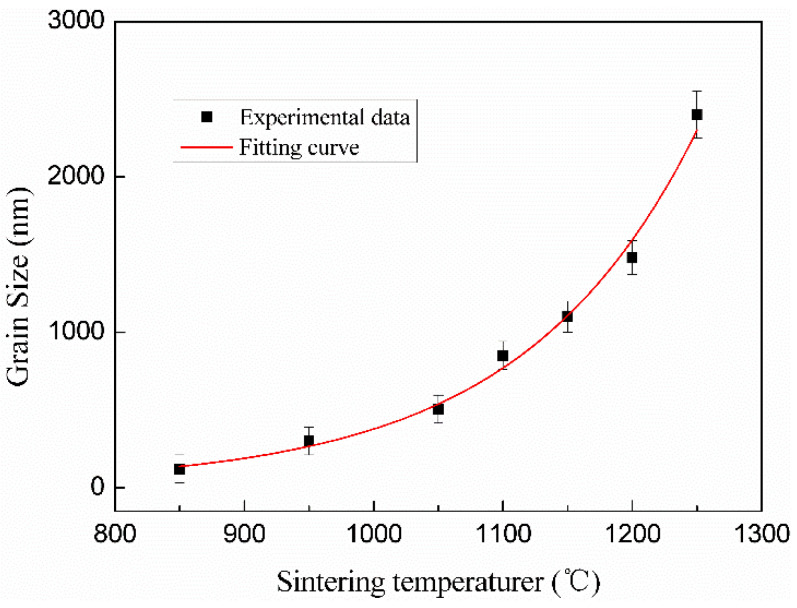
Grain size variation curve of pure HA prepared with different sintering temperatures for 2 h.

In terms of pure HA and the ZnO/HA composite in the study, experimental results have shown that there are mainly three factors that contribute to compression strength: I densities; II grain size of HA; III morphology, size, and distribution of the second-phase particles. As shown in Figure 8, it was the grain size variation of pure HA at different sintering temperatures for 2 h. The experimental results showed that the grain size of HA became big gradually with the increase in temperature, and the variation rule after fitting can be described by Equation (1). It has been found that the fracture mode of HA is mainly transgranular fracture. The experiment results also indicate that the density increased with the growth of the grains. In addition, the grain growth reduced the area of the fracture crystal face, which was contrary to the contribution of increasing density to the compressive strength of materials. That is why the maximum value of compressive strength appeared at 1150 °C. For ZnO/HA composites, the number of internal pores increased with the addition of Zn, and there was a smaller contribution on the improvement in the density with the increase in sintering temperature and extension of holding time. It can be inferred that the positive factors on the improvement in the compressive strength are mainly II and III.

(1) $$y=y_{0}+a\times \left(e^{\left(\frac{x-b}{c}\right)}-1\right)$$

where y is the grain size; y_0_ is the original grain size; x is the sintering temperature; a, b, and c are three constants.

**Figure 9:**
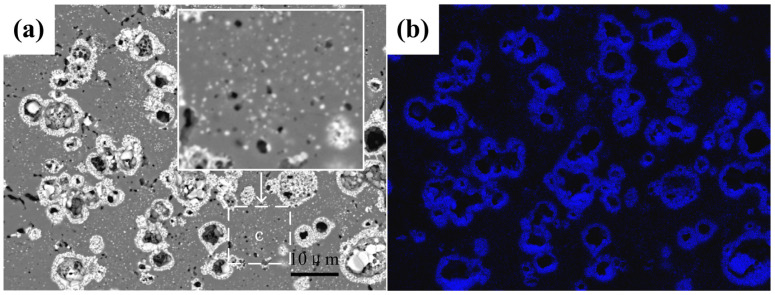
(**a**) SEM morphology and (**b**) EDS elemental map of Zn element distribution map of ZnO/HA composite sample with 20% Zn addition sintered at 1250 °C for 2 h.

ZnO, as the second phase, existed mainly in two size distributions in the materials: I formed by oxidation based on Zn elemental particles, was large in number and concentrated, and the grain size of ZnO was also greatly affected by temperature; II fine grain size ZnO has been described in point c in Figure 5a. These fine ZnO particles hinder the movement of HA grain boundaries around them (such as the area of Figure 9b). The fracture mode of HA was transgranular fracture mainly (cleavage fracture), which indicates that the cohesive energy of the HA grain boundary was greater than that of the cleavage fracture surface. Therefore, grain refinement increased the grain boundary area and hindered the propagation of cracks. At the same time, the orientation of adjacent HA grains was randomly distributed, leading to the need to change the direction of cracks when they propagated from the interior of adjacent grains of one grain phase (ZnO second phase played this role as well). Therefore, the increases in this effect (as shown in the region of Figure 9c) increased the area of the fracture surface and the compressive strength.

## 4. Conclusions

The n-HA powders were successfully prepared by the chemical precipitation method, and Zn particles could be used as a pore forming agent material to prepare porous HA composites with controllable shape, size, and distribution. HA grain orientation was randomly distributed in ZnO/HA composites. Oxidation of Zn took place during the sintering process, which was in three stages: Solid phase, liquid phase, and gas phase. Fine ZnO particles were formed and distributed between the HA grains during the stages of liquid and gas, which hindered HA grain boundary movement and led to the refinement of HA. The HA grains were randomly distributed, and the crack propagation was hindered by the grain boundary and the second-phase particles (ZnO). The direction of crack propagation, when it expands to other grains, needed to be changed, which increased the area of the cleavage fracture surface and improved the mechanical properties of the composites. The sample with the amount of 20% zinc had the best mechanical properties. The compression strength of ZnO/HA composites was 187% higher than that of pure HA, and the mechanical properties were the best of the sample containing 20% zinc. Controlling the size and distribution of the second-phase particles is effective to control the mechanical properties of ZnO/HA composites. 

## Figures and Tables

**Table 1:** Information of experimental equipment for the preparation.

Equipment	Product Model	Manufacturer
Analytical balance	FA2004	Shanghai Tianping instrument Factory, Shanghai, China
Electric mixer	JJ-1	Jiangsu Jintan Jincheng Guosheng Experimental Instrument Factory, Changzhou, China
Constant temperature drying oven	101A-2	Shanghai Experimental Instrument General Factory, Shanghai, China
Constant temperature water bath	DK-98-IIA	Tianjin Tester Instrument Co. LTD, Tianjin, China
Mixer	4 Tank Mixer	MTI corporation
Powder compressing machine	ZHY-601B	Beijing Zhonghe Venture Technology Development Co. LTD, Beijing, China
Hydroextractor	LD5-A	Beijing Medical centrifuge Plant, Beijing, China
Resistance furnace	SX2-5-12G	Jinan Precision Science instrument Co. LTD, Jinan, China

**Table 2:** Composition and sintering process parameters (Bal., the mass fraction except for the other ingredient) of pure HA and ZnO/HA composites.

Experimental Group	Mass Fraction/wt.%		Prefabricated Pressure (MPa)	Sintering Temperature (°C)	Sintering Time (h)
	HA	Zn			
1	Bal.	0	150	850	2
2	Bal.	0	150	950	2
3	Bal.	0, 10, 20, 30	150	1050	2
4	Bal.	0, 10, 20, 30	150	1100	2
5	Bal.	0, 10, 20, 30	150	1150	2
6	Bal.	0, 10, 20, 30	150	1200	2
7	Bal.	0, 10, 20, 30	150	1250	2
